# Clinical Characteristics and Microorganisms Isolated in Community-Acquired Pneumonia in the COVID-19 Period

**DOI:** 10.1155/2024/5948747

**Published:** 2024-03-19

**Authors:** Meritxell Gavalda, Maria Isabel Fullana, Adrià Ferre, Rebecca Rowena Peña, Julen Armendariz, Orla Torrallardona, Aina Magraner, Alejandro Lorenzo, Carles García, Gemma Mut, Lluís Planas, Carla Iglesias, Pablo Fraile-Ribot, Maria Dolores Macia Romero, Melchor Riera, Mercedes García-Gasalla

**Affiliations:** ^1^Internal Medicine Department, Hospital Universitari Son Espases, Illes Balears, Palma, Spain; ^2^Microbiology Department, Hospital Universitari Son Espases, Illes Balears, Palma, Spain; ^3^Fundació Institut d'Investigació Sanitària Illes Balears (IdISBa), Illes Balears, Palma, Spain; ^4^Universitat de les Illes Balears, Illes Balears, Palma, Spain

## Abstract

**Introduction:**

Community-acquired pneumonia is a leading cause of mortality and hospital admissions. The aetiology remains unknown in 30–65% of the cases. Molecular tests are available for multiple pathogen detection and are under research to improve the causal diagnosis.

**Methods:**

We carried out a prospective study to describe the clinical characteristics and aetiology of community-acquired pneumonia during the COVID-19 pandemic and to assess the diagnostic effectivity of the microbiological tests, including a molecular test of respiratory pathogens (FilmArray™ bioMérieux).

**Results:**

From the 1st of February 2021 until the 31st of March 2022, 225 patients were included. Failure in microorganism identification occurred in approximately 70% of patients. *Streptococcus pneumoniae* was the most common isolate. There were 5 cases of viral pneumonia. The tested FilmArray exhibited a low positivity rate of 7% and mainly aided in the diagnosis of viral coinfections.

**Conclusions:**

Despite our extensive diagnostic protocol, there is still a low rate of microorganism identification. We have observed a reduction in *influenza* and other viral pneumoniae during the COVID-19 pandemic. Having a high NEWS2 score on arrival at the emergency department, an active oncohematological disease or chronic neurological conditions and a positive microbiological test result were related to worse outcomes. Further research is needed to determine the role of molecular tests in the microbiological diagnosis of pneumonia.

## 1. Introduction

Community-acquired pneumonia is a leading cause of hospital admissions and mortality. Its incidence in adults ranges between 3 and 20 cases per 1000 inhabitants per year [1]. Before the COVID-19 pandemic, it caused approximately 23,000 annual deaths in the European Union, corresponding to 6% of total mortality [2]. About 30–65% of the causes of pneumonia remain unknown [3, 4]. Finding the cause of pneumonia can be useful in various situations. It can help detect resistant pathogens, guide antibiotic therapy, recognise public health implications for certain pathogens like *Legionella*, adjust initial empirical therapy when it fails, and track changes in the epidemiology of pneumonia [5, 6].

Several studies have demonstrated the prognostic value of identifying the cause of pneumonia. For example, Uematsu et al. found that following microbiological testing guidelines (sputum, blood cultures, and urine antigen tests) on the first day of hospitalization was associated with lower mortality and shorter hospital stays [7]. Similarly, Costantini et al. demonstrated that performing guideline-recommended urinary antigen tests was associated with lower odds of in-hospital mortality and 30-day mortality [8]. Fullana Barceló et al. found that a delayed diagnosis of influenza infection was related to clinical complications [9]. However, some studies have failed to prove the prognostic value of microbiological testing [10–13], although these studies focused on a single microbiological test (sputum, urinary antigen, or blood culture) and not combined testing.

The 2019 IDSA guidelines recommend obtaining sputum cultures, blood cultures, and *Legionella* and *Pneumococcal* urinary antigen tests in cases of severe pneumonia. Additionally, they recommend obtaining sputum and blood cultures for inpatients empirically treated for MRSA or *Pseudomonas aeruginosa* [5]. However, this recommendation is based on low quality of evidence. For this reason and with the aim of enabling targeted therapies, the guidelines encourage new research to find rapid, cost-effective, sensitive, and specific diagnostic tests that identify the causative microorganism.

In recent years, molecular tests have become available for multiple pathogen detection. They are the preferred method for diagnosing some pathogens, such as viruses and *Mycoplasma pneumoniae* [14]. Molecular tests optimize antibiotic selection and allow for de-escalation and for the reduction of antibiotic and antifungal use in viral pneumonia [15]. These techniques have also proven to shift pneumonia aetiology, with a higher prevalence of viruses and atypical microorganisms [16–18].

This study has two main objectives. The first is to describe the clinical characteristics and aetiology of community-acquired pneumonia during the COVID-19 pandemic. The second is to assess the diagnostic effectivity of the microbiological tests, including a molecular test of respiratory pathogens (FilmArray™ bioMérieux) performed as a nasal swab.

## 2. Methods

**Figure 1:**
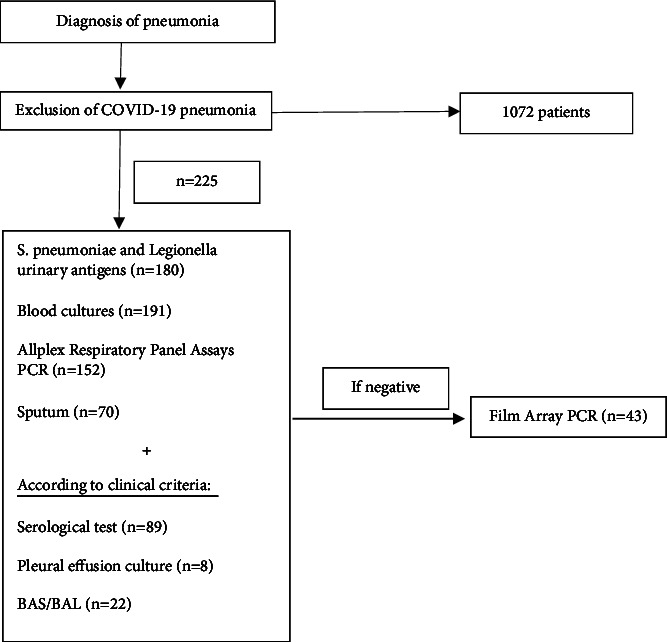
Flowchart for pneumonia diagnosis.

This study has a prospective design: all patients admitted for community-acquired pneumonia (except COVID-19 cases) collected from the 1st of February 2021 until the 31st of March 2022. The protocol for respiratory infections in our hospital includes the following: blood cultures, sputum cultures (when possible), *Legionella* and *Pneumococcal* urinary antigen testing, and viral nasal swab (Allplex Respiratory Panel Assays PCR) in all patients. Allplex Respiratory Panel Assays PCR includes testing for FluA, FluA-H1, FluA-H1pdm09, FluA-H3, FluB, RSV-A, RSV-B; AdV, HEV, MPV, PIV-1, PIV-2, PIV-3, PIV-4, HBoV, human coronavirus 229E, NL63, OC43, and HRV. In the cases for which all tests were negative, we added our novel test: the respiratory pathogens FilmArray. This test includes identification for coronavirus 229E, coronavirus HKU1, coronavirus OC43, coronavirus NL63, metapneumovirus, rinovirus/enterovirus, influenza A, influenza A/H1, influenza A/H1-2009, influenza A/H3, influenza B, parainfluenza 1, parainfluenza 2, parainfluenza 3, parainfluenza 4, VRS, *Bordetella pertussis*, *Chlamydophila pneumoniae*, and *Mycoplasma pneumoniae*.  Figure 1 represents the protocol that was followed.

According to clinical criteria, other microbiological tests were performed: *Mycoplasma pneumoniae*, *Chlamydophila pneumoniae*, *Coxiella burnetti*, *Chlamydophila psittaci* serological tests, bronchoalveolar lavage or aspirate, and culture of pleural effusion. Immunofluorescence is used for *C. psittaci* (IgM and IgG) and *C. burnetti* (IgM, IgG, and phases) while *M. pneumoniae* (IgM e IgG) are analysed by automated CLIA technique. For the serological tests, a confirmed diagnosis needed to meet the following criteria: (a) 2 serological tests 2–4 weeks apart confirming a 4-time elevation of IgG titers or (b) 2 serological tests 2–4 weeks apart with seroconversion (the first with positive IgM and negative IgG and the second with positive IgG). A possible diagnosis is given when the following criteria were met: (a) only 1 serological result available with a positive IgM and a negative IgG or (b) elevated IgG titles suggesting acute infection (ie IgG >1/1024 for *Chlamydophila pneumoniae*). Patients included in the study signed an informed consent form.

Data collection included age, sex, comorbidities, NEWS2 score at admission, infection markers in blood tests, microbiological tests performed, and clinical evolution during admission (ICU admission, death, and discharge). Categorical variables were expressed as numbers and percentages, and continuous variables as mean and standard deviation (SD) or median and percentiles p25 and p75 if a nonnormal distribution was found. Proportions for categorical variables were compared using the *χ*^2^ test. The independent group Mann–Whitney *U* test was used for the comparison of continuous variables. All statistical analyses were performed using SPSS (Statistical Package for the Social Sciences) version 22.0 software (SPSS Inc.). Two-sided *p* values of less than 0.05 were considered statistically significant.

## 3. Results

In total, 225 patients were included in the study. Patients admitted for COVID-19 pneumonia (a total of 1072 during the study period) were excluded. Epidemiological data and comorbidities are presented in Table 1.

The median NEWS2 score on arrival at the emergency room was 4 points [2–6.5]. The blood test showed median leukocytes of 13,4000 [9,200–17,000] with C reactive protein values of 10.8 mg/dl [4.3–22.4] and procalcitonin of 0.4 ng/dl [0.1–1.6]. A wide majority of patients (168, 74.67%) had lobar pneumonia. However, 41 (18.22%) had a radiologically bilobar pneumonia, and 13 (5.78%) had a multilobar condensation or infiltrate.

Concerning the results of diagnostic tests: urinary antigen testing was performed in 180 of the patients (80%) and was positive in 20 (11.1%); 15 of the positives were for *S. pneumoniae* and the rest for *L. pneumophila*. Blood cultures were obtained in 191 patients (84.89%), and 13 (6.8%) were positive. Viral PCR was conducted in 152 patients (67.56%) and was positive in 23 instances (15.1%). Sputum cultures were obtained in 70 patients (31.11%), and 21 of them were positive (30%). Serological tests for atypical pneumonia were performed in 89 patients and were positive in 35 cases, but only 3 met the criteria for a confirmed diagnosis due to the absence of a second serological test. The FilmArray PCR was performed in 43 patients with 3 positive results. Pleural effusion analysis and culture were performed in 8 patients (3.56%) and yielded 2 (25%) positive results. Bronchial aspirate (BAS) or bronchoalveolar lavage (BAL) was obtained for 22 (9.78%) of the patients, and 9 were positive (40.91%).

Despite the above-described testing, no causal agent was identified in most patients (157, 69.77%). Of the patients for whom an aetiological diagnosis could be obtained, *Streptococcus pneumoniae* was the most common isolate [19] followed by *Pseudomonas aeruginosa* [7]. Aspiration pneumonia was suspected in 44 patients (19.56%) by the clinician (Table 2).

Concerning patient outcomes, 14 (6.2%) were admitted to the ICU, 11 of which required endotracheal intubation. 7 patients required high-flow nasal oxygen cannula at some point. The median admission time was 7 days [5–12], and the median antibiotic course was 9 days [7–12]. Mortality during admission was 12.4%.

We studied the differences between patients with pneumonia of known versus unknown aetiology. In the unknown aetiology group, there were more cases of bronchoaspiration, shorter antibiotic courses, a lower rate of ICU admissions, and shorter hospital admissions (Table 3).

We also searched for differences between patients who died during admission and those who did not. The patients who died during admission had a higher NEWS2 score, were older, had a higher Charlson score, had a higher prevalence of active oncohematological disease and chronic neurological disease, and had a higher rate of bronchoaspiration, a longer antibiotic course, and a greater need of high flow oxygen cannula (Table 4).

## 4. Discussion

Our study revealed several important findings. First, we observed a low rate of microorganism identification in our patient cohort despite our extensive diagnostic protocol (including FilmArray for respiratory pathogens). Failure in microorganism identification occurred in approximately 70% of patients. This finding is consistent with previous studies in the existing literature [3, 4]. Notably, we found that cases with unknown aetiology were more common in patients with shorter hospital admissions, which likely limited the ability to conduct comprehensive diagnostic tests.

Similar to the findings in other studies in North America, Europe, and Australia [19, 20], *Streptococcus pneumoniae* was the most frequently identified pathogen in our series. The second most frequent microorganism was *Pseudomonas aeruginosa*, typically associated with nosocomial pneumonia. The proportion of *Pseudomonas aeruginosa* cases identified was similar to that reported in other studies [21]. *Klebsiella pneumoniae*, *E. coli*, and other GNB, frequently isolated in patients with pneumonia in tropical areas, notably in Southeast Asian countries, are found in a small proportion of patients in our series and in other Western world series [22, 23].

Remarkably, there were very few cases of viral pneumonia during the study period, excluding those caused by COVID-19. The total number of viral pneumoniae amounted to only 5 cases, including 3 cases of *metapneumovirus* and 2 cases of *respiratory syncytial virus.* No instances of influenza-related pneumonia were observed during the study period, which was consistent with the flat curve of influenza incidence in the Balearic Islands [24]. We hypothesize that the widespread use of masks, adherence to social distancing measures, and a higher rate of influenza vaccination likely contributed to the reduction in viral pneumonia cases. In a prior study with a higher positive rate in aetiology, viral pneumonia was identified as a significant cause [25]. However, in our current series, the prevalence of viral pneumonia was notably low, attributed to the unique epidemiologic situation during the COVID-19 pandemic. This fact has probably contributed to a lower diagnosis in our study.

We found no significant association between microorganism identification and mortality. On the contrary, patients with an unknown aetiology had shorter courses of antibiotic treatment and hospital stays. This may suggest a greater ease in identifying the causative microorganism in more severe forms of the disease for reasons yet to be determined. Therefore, the possibility of isolating the causative microorganism could be used as a marker of disease severity, as noted in other studies [26].

In our study, bronchoalveolar aspirate or lavage was identified as the test with the highest predictive positive value (40%), especially in patients with severe pneumonia. It is also noteworthy that patients with shorter stays did not undergo serological testing, which may have limited the breadth of diagnostic possibilities. Consequently, patients with prolonged hospital stays and more severe pneumonia were subjected to a more extensive battery of tests, thereby increasing the likelihood of obtaining a comprehensive aetiological diagnosis.

In a previous study, patients with unknown aetiology had worse outcomes [25]. However, this is most likely because most patients died before 48 hours of admission due to delayed or lack of ICU care therefore limiting the possibility of an ethology work-up.

Our study aimed to assess the performance of diagnostic tests, with sputum analysis and viral PCR emerging as the most useful ones. However, sputum analysis has limitations as it requires patient cooperation, leading to its successful processing in only 31% of cases. Moreover, most sputum samples were collected postantibiotic initiation due to challenges in obtaining them upon admission to the emergency room. On the other hand, viral PCR yielded positive results in 23 patients, primarily contributing to the identification of coinfections (*Rhinovirus*, *Adenovirus*, or *Enterovirus*). They were the primary cause of pneumonia in only 5 cases.

We also evaluated the performance of a molecular test for respiratory pathogens (FilmArray™ bioMérieux) conducted using a nasal swab. However, this test exhibited a low positivity rate of 7% among the tested patients and mainly aided in the diagnosis of coinfections (1 case of diagnostic *respiratory syncytial virus* and 2 cases of coinfection with *rhinovirus/adenovirus*). Nevertheless, further investigation is warranted to explore the potential of these novel techniques, as other studies have proven their ability to improve diagnosis and treatment outcomes [27]. There is improvement in the diagnosis rates in lower respiratory tract samples, but the beneficial effect on nasopharyngeal samples is uncertain due to possible contamination [28–31].

Our study had some limitations. Not all tests were performed on every patient, especially the molecular test for respiratory pathogens. In most cases, only one serological test was performed instead of two, which potentially might have underestimated the diagnosis of atypical pneumoniae. Additionally, we were unable to establish a correlation between the identification of aetiology and improved prognosis in community-acquired pneumonia, possibly due to sample size constraints.

In conclusion, we have observed a reduction in influenza and other viral pneumoniae during the COVID-19 pandemic. Despite our extensive diagnostic protocol, there is still a low rate of microorganism identification. Having a high NEWS2 score on arrival at the emergency department, an active oncohematological disease or chronic neurological conditions and a positive microbiological test result should serve as an alarm for clinicians, prompting them to provide enhanced attention to the patient. Furthermore, further research is needed to determine the role of molecular tests in the microbiological diagnosis of pneumonia.

## Figures and Tables

**Table 1:** Demographic data.

Demographic characteristic	Total (%) *n* = 225
Sex (male), *n* (%)	133 (58.7%)
Age, years [median (percentiles 25–75)]	73 (61–85)
Smoker status, *n* (%)	40 (17.8%) current smokers
	84 (37.3%) used to smoke
Charlson score, points [median (percentiles 25–75)]	5 (3–7)
High blood pressure, *n* (%)	135 (60%)
Diabetes mellitus, *n* (%)	58 (25.8%)
Chronic obstructive pulmonary disease, *n* (%)	57 (25.3%)
Asthma, *n* (%)	15 (5.75%)
Chronic heart failure, *n* (%)	68 (30.2%)
Chronic kidney disease, *n* (%)	59 (26.2%)
Chronic neurologic disease, *n* (%)	21 (9.3%)
Active neoplastic disease, *n* (%)	21 (9.3%)
Immunosuppressed, *n* (%)	24 (10.7%)
HIV, *n* (%)	6 (2.7%)

**Table 2:** Aetiologies.

Microorganism	Number (%)
Unknown	157 (69.7%)
*S. pneumoniae*	19 (8.4%)
*Pseudomonas aeruginosa*	7 (3.1%)
*Legionella*	5 (2.25)
*Klebsiella pneumoniae*	4 (1.8%)
*E. coli*	4 (1.8%)
MRSA	3 (1.3%)
Metapneumovirus	3 (1.3%)
Respiratory syncytial virus	2 (0.9%)
*Haemophilus influenzae*	2 (0.9%)
*Coxiella burnetii*	2 (0.9%)
*S. gallolyticus*	2 (0.9%)
Coinfection	6 (2.7%)

Coinfection cases included. 1. *E. coli*, *K. pneumoniae*, and *P. aeruginosa*. 2. *K. pneumoniae* and *E. coli*. 3. *S. constellatus* and MSSA. 4. Rhinovirus and Citrobacter. 5. Rhinovirus and possible *M. pneumoniae*. 6. Rhinovirus and possible *C. pneumoniae*.

**Table 3:** Differences between patients with known etiology and unknown etiology.

	Known etiology (*n* = 68)	Unknown etiology (*n* = 157)	*p* value
Age, years [median (percentiles 25–75)]	72 (62–83)	75 (61–86)	0.232
Sex (male)	42 (62%)	90 (57%)	0.535
HBP, *n* (%)	36 (53%)	99 (63%)	0.155
DM, *n* (%)	13 (19%)	45 (29%)	0.133
COPD, *n* (%)	22 (32%)	35 (22%)	0.111
Asma, *n* (%)	2 (3%)	13 (8%)	0.242
Chronic heart disease, *n* (%)	18 (27%)	50 (32%)	0.420
Chronic renal disease, *n* (%)	7 (10%)	31 (20%)	0.082
Chronic neurological disease, *n* (%)	13 (19%)	46 (29%)	0.111
Chronic hepatic disease, *n* (%)	6 (9%)	11 (7%)	0.636
Active oncohematological disease, *n* (%)	8 (12%)	13 (8%)	0.409
Immunocompromised patients, *n* (%)	8 (12%)	16 (10%)	0.725
HIV, *n* (%)	5 (3%)	1 (1.5%)	0.671
Charlson score [median (percentiles 25–75)]	5 (3–6)	5 (3–7)	0.360
Leucocytes (×10^3^) [median (percentiles 25–75)]	13.9 (10–18.6)	13.2 (0.2–16.8)	0.392
PCR (*n* = 220) [median (percentiles 25–75)]	15.3 (5.5–25.3)	10.3 (4.2–20.9)	0.075
PCT (*n* = 70) [median (percentiles 25–75)]	0.6 (0.11−2.69)	0.24 (0.12−1)	0.261
NEWS2 score [median (percentiles 25–75)]	4 (2–7)	4 (3–6)	0.621
Antibiotic duration (days)	11 (8–16)	9 (7–11)	**0.000**
Admission duration (days)	10 (6–16)	7 (4–11)	**0.004**
Bronchoaspiration, *n* (%)	7 (10%)	37 (24%)	0.021
ICU, *n* (%)	11 (16%)	3 (2%)	**0.000**
Intubation, *n* (%)	8 (12%)	3 (2%)	**0.004**
Intubation duration (days), *n* (%)	7 (2–9)	2 (1–3)	0.222
High flow oxygen, *n* (%)	2 (3%)	5 (3%)	1.000
High flow oxygen duration (days)	1	4 (2–7)	0.267
Mortality, *n* (%)	9 (13%)	19 (12%)	0.813
Transfer to sociosanitary facility, *n* (%)	6 (9%)	17 (11%)	0.676

The bold values indicate that statistical significance was set at *p* < 0.05.

**Table 4:** Differences between patients with hospital mortality and patients alive at discharge.

	Deceased (*n* = 28)	Alive (*n* = 197)	*p* value
Age, years [median (percentiles 25–75)]	80 (72–89)	73 (61–85)	**0.009**
Sex (male)	17 (61%)	115 (58%)	0.814
HBP, *n* (%)	18 (64%)	117 (59%)	0.621
DM, *n* (%)	8 (29%)	50 (25%)	0.718
COPD, *n* (%)	8 (29%)	49 (25%)	0.674
Asma, *n* (%)	2 (7%)	13 (7%)	1
Chronic heart disease, *n* (%)	8 (29%)	60 (31%)	0.839
Chronic renal disease, *n* (%)	4 (14%)	34 (17%)	1
Chronic neurological disease, *n* (%)	12 (43%)	47 (24%)	0.032
Chronic hepatic disease, *n* (%)	1 (4%)	16 (8%)	0.702
Active oncohematological disease, *n* (%)	6 (21%)	15 (8%)	0.031
Immunocompromised patients, *n* (%)	4 (14%)	15 (8%)	0.513
HIV, *n* (%)	1 (4%)	5 (2.5%)	0.554
Charlson score [median (percentiles 25–75)]	6 (4–8)	5 (3–6)	**0.036**
Leucocytes (×10^3^) [median (percentiles 25–75)]	13.5 (10.3–20.2)	13.3 (9.2–17)	0.385
PCR (*n* = 220) [median (percentiles 25–75)]	16.5 (3.5–24.9)	10.7 (4.5–22.2)	0.543
PCT (*n* = 70) [median (percentiles 25–75)]	2.3 (0.16–5.43)	0.31 (0.1–1.21)	0.101
NEWS2 score [median (percentiles 25–75)]	7 (4–9)	4 (2–6)	**0.001**
Antibiotic duration (days)	7 (4–11)	10 (7–12)	**0.004**
Admission duration (days)	7 (3–16)	7 (5–12)	0.861
Bronchoaspiration, *n* (%)	13 (47%)	31 (16%)	**0.000**
ICU, *n* (%)	3 (11%)	11 (6%)	0.392
Intubation, *n* (%)	3 (11%)	8 (4%)	0.144
Intubation duration (days), *n* (%)	5 (2–7)	4 (2–9)	0.667
High flow oxygen, *n* (%)	3 (11%)	4 (2%)	0.043
High flow oxygen duration (days)	3 (1–8)	1 (1–5)	0.7
Transfer to sociosanitary facility, *n* (%)	1 (4%)	22 (11%)	0.324

The bold values indicate that statistical significance was set at *p* < 0.05.

## Data Availability

The data used to support the findings of this study are included within the article.
